# Expression of *p53* N-terminal isoforms in B-cell precursor acute lymphoblastic leukemia and its correlation with clinicopathological profiles

**DOI:** 10.1186/s12885-020-6599-8

**Published:** 2020-02-10

**Authors:** Lixian Oh, Pierre Hainaut, Sandrine Blanchet, Hany Ariffin

**Affiliations:** 1 Department of Paediatrics, Faculty of Medicine, University of Malaya, Kuala Lumpur, Malaysia; 2 Institute of Advanced Biosciences, INSERM 1209 CNRS 5309 University of Grenoble-Alpes, Grenoble, France

**Keywords:** Childhood ALL, p53 tumour suppressor protein, Protein isoforms

## Abstract

**Background:**

*TP53* mutations occur in only about 3% of primary and 10–20% of relapse B-cell precursor acute lymphoblastic leukaemia (BCP-ALL). However, alternative mechanisms may contribute to functionally impairing the p53 pathway in the absence of a mutation. Candidate mechanisms include overexpression of p53 mRNA variants encoding either dominant-negative p53 protein isoforms such as Delta40p53 and Delta133p53, or modulatory isoforms such as p53beta, which counteract the effects of Delta133p53 on replicative senescence in T-lymphocytes.

**Methods:**

We used semi-quantitative reverse-transcriptase PCR (RT-PCR) and Western blot to investigate the expression of full length p53 (TAp53), Delta40p53, Delta133p53 or p53beta in diagnostic marrow from a clinical cohort of 50 BCP-ALL patients without *TP53* mutation (29 males and 21 females, age range 2–14 years) and in the bone marrow cells of 4 healthy donors (used as controls).

**Results:**

Irrespective of isoforms, levels of p53 mRNA were low in controls but were increased by 2 to 20-fold in primary or relapse BCP-ALL. TAp53 was increased in primary BCP-ALL, Delta40p53 was elevated in relapse BCP-ALL, whereas Delta133p53 and p53beta were increased in both. Next, mRNA levels were used as a basis to infer the ratio between protein isoform levels. This inference suggested that, in primary BCP-ALL, p53 was predominantly in active oligomeric conformations dominated by TAp53. In contrast, p53 mostly existed in inactive quaternary conformations containing ≥2 Delta40 or Delta133p53 in relapse BCP-ALL. Western blot analysis of blasts from BCP-ALL showed a complex pattern of N-terminally truncated p53 isoforms, whereas TAp53beta was detected as a major isoform. The hypothesis that p53 is in an active form in primary B-ALL was consistent with elevated level of p53 target genes *CDKN1A* and *MDM2* in primary cases, whereas in relapse BCP-ALL, only *CDKN1A* was increased as compared to controls.

**Conclusion:**

Expression of p53 isoforms is deregulated in BCP-ALL in the absence of *TP53* mutation, with increased expression of alternative isoforms in relapse BCP-ALL. Variations in isoform expression may contribute to functional deregulation of the p53 pathway in BCP-ALL, specifically contributing to its down-regulation in relapse forms.

## Background

Acute lymphoblastic leukaemia (ALL) is the commonest childhood cancer. ALL accounts for more than 75% of childhood leukaemia, occurring more frequently in B- lineage than T-lineage, with a peak prevalence in children aged 3 to 5 years. The current WHO classification of B-cell precursor ALL (BCP-ALL) is largely based on molecular and cytogenetic features. It identifies up to 9 subtypes, characterized by either specific chromosomal translocations or rearrangements, and by ploidy status [1]. These subtypes are associated with different therapeutic responses and risks of disease progression. Current treatment protocols use risk-stratified, multi-agent chemotherapy, leading to a cure rate of about 90% in high-income countries. However, despite initial treatment response, disease relapse occurs in 10–15% of patients and is one of the leading causes of cancer mortality in children [2].

Mutations in the *TP53* suppressor gene are rare events in BCP-ALL, occurring in only 3% of primary cases [3]. Mutations are not equally distributed among BCP-ALL subtypes. In particular, pathogenic germline *TP53* variants are identified in about 65% of low hypodiploid BCP-ALL [4]. In subtypes other than low hypodiploid, somatic mutations are detected in about 12–20% of relapses, 4 to 7 times more frequent than in primary ALL [4]. These observations suggest that, with the exception of low hypodiploid forms, most cases of BCP-ALL develop in the presence of a potentially functional *TP53* gene. Given the multiple roles of this master suppressor in controlling anti-proliferative responses encompassing cell cycle arrest, apoptosis, differentiation, senescence, DNA repair, metabolism and immune response [5], the low frequency of somatic *TP53* mutations in BCP-ALL raises the question whether the p53 pathway might be functionally impaired by mechanisms other than mutations in these neoplasms.

Alternative mechanisms of p53 protein inactivation have been identified in many types of cancer. They include targeting and increased degradation of wild-type p53 by viral antigens in the case of cervical and oral cancers caused by oncogenic HPV forms [6] or over-expression of MDM2/MDM4 and degradation of p53 in several cancers including sarcomas and melanomas [7–9]. In addition, p53 mRNA is targeted by several microRNAs and it has been suggested that increased microRNAs expression may contribute to attenuate wild-type p53 expression [10]. In particular, miR125b, which targets the 3’UTR of p53 mRNA, is overexpressed in several haematological malignancies including BCP-ALL and shows leukaemogenic properties when overexpressed in mouse B-cells [11]. However, it is not known whether these oncogenic effects are caused by down-regulation of p53-mRNA.

Another putative mechanism of functional inactivation of p53 is the overexpression of p53 mRNA variants encoding dominant-negative p53 protein isoforms, including variants lacking parts encoding the p53 protein N-terminus that contain the main transactivation domain of p53. The resulting protein isoforms Delta40p53 and Delta133p53 lack the first 39 and 132 residues, respectively and have been shown to dominantly inhibit p53 function when overexpressed in the presence of wild-type p53 [12–14]. These isoforms are generated either by alternative splicing or internal AUG codon usage (Delta40p53) and by alternative promoter usage (Delta133p53). Other p53 isoforms have been identified, in which parts of the C-terminus of p53 (containing, among others, the oligomerization domain) are replaced by short, specific amino-acid sequences encoded by alternatively-retained *TP53* introns 9. These isoforms include p53beta, in which the 62 C-terminal residues are replaced by 10 amino-acids encoded from intron 9 [14]. Interestingly, Delta133p53 and p53beta have been shown to exert opposite effects in the regulation of replicative senescence of human cells in vitro, including T-lymphocytes. Whereas high expression of Delta133p53 is associated with sustained proliferative potential, the expression of p53beta increases in cells that undergo replicative senescence [15]. Therefore, it is possible that changes in p53 protein isoforms may play an important role in, controlling the proliferation of various human cell times in specific phases of their proliferation/differentiation trajectories.

In this study, we analysed the mRNA and protein expression of full-length p53 protein (TAp53), Delta40p53, Delta133p53 and p53beta in diagnostic bone marrow samples from 50 patients with BCP-ALL without *TP53* mutation and compared these findings with those from 4 healthy donor marrows. We sought to understand whether changes in the expression of p53 isoforms regulate the p53 pathway in BCP-ALL, specifically contributing to its down-regulation in relapse forms.

## Methods

### Patients and sample collection

Bone marrow samples were collected at diagnosis (Day 0) from 50 children diagnosed with de novo (*n* = 40) or relapse (*n* = 10) BCP-ALL at the Department of Paediatrics of University of Malaya Medical Centre, Kuala Lumpur, Malaysia and from 4 healthy sibling donors of paediatric bone marrow transplant recipients excess following transplantation. Samples were anonymized and all patients or legal guardians gave written informed consent to have their tissue and clinical data used in this study in accordance to the Helsinki Declaration. The study was reviewed and approved by the Institutional Review Board of University Malaya Medical Centre (MEC 2016922–4275). Diagnosis was established according to morphological examination and immunophenotyping. In subjects with de-novo BCP-ALL, 10 were positive for *BCR-ABL1*, 1 for *ETV6-RUNX1*, 10 had hyperdiploidy (chromosomes> 50) and 19 were negative for tested molecular/cytogenetic features. Mononuclear cells from the bone marrow (termed BMMCs) were separated through density gradient centrifugation (Ficoll-Paque Plus, GE Healthcare Bioscience, USA) and washed 3 times with 1X HBSS (Hank’s Balanced Salt Solution, ThermoFischer Scientific, USA). The cell pellet was suspended in either (1) Trizol® (Thermo Fischer Scientific, USA) at 10 million cells/1 mL for RNA extraction or (2) in RIPA buffer (Intron Biotechnology, Korea) containing 1% protease inhibitor (Thermo Fischer Scientific, USA) and 1% phosphatase inhibitor (Thermo Fischer Scientific, USA) at 10 million cells/50uL for protein extraction.

### Semi-quantitative RT-PCR

Semi-quantitative RT-PCR analysis was performed with Qiagen RotorgeneQ Real-time PCR system using SYBR-Green detection according to manufacturer’s instructions. Briefly, total RNA was extracted from BMMCs using the Trizol®/Qiagen RNeasy hybrid RNA extraction kit (Qiagen, Germany) and the RNase-free DNase-treatment kit (Qiagen, Germany). 1μg of the obtained DNA-free RNA was reverse-transcribed using Omniscript® RT Kit (Qiagen, Germany). Each PCR assay was performed in a 20uL reaction containing QuantiNova SYBR Green PCR mix (Qiagen, Germany), primers specific for each transcript (Additional file 1) and 3uL of 5-fold diluted cDNA (equivalent to 30 ng). From a panel of 10 reference genes, *RPL27*, *CYC1* and *RNU6–1* were selected as the most stable using geNorm, Normfinder and BestKeeper. The sequences and location of all primers are presented in Additional file 1. Each gene analysis was performed in triplicate and real-time PCR products were verified by melting curve analysis. Cq (threshold cycle) was determined for each assay (software) and Cq value > 35 was interpreted as non-expressed. The relative expression of p53 isoform transcripts was normalized to a geometric average expression level of the 3 reference genes and expressed as the fold change to calibrator (healthy marrow) calculated using Delta-Delta Cq method.

### Western immunoblotting

Total protein lysate was extracted from BMMCs using RIPA buffer (Intron Biotechnology, Korea) containing a cocktail of 1% phosphatase- and 1% protease inhibitors (Thermo Fischer Scientific, USA). Sodium dodecyl sulphate-polyacrylamide gel electrophoresis and electroblotting were performed using anti-p53 (9282, Cell Signaling Technology, US and DO-7 sc-47,698 Santa Cruz Biotechnology, US), anti-MDM2 (MABE281, Merck Millipore, US), and anti-p21^WAF-1^ (OP64, Merck Millipore, US) antibodies. Equal protein loading was assessed using anti-alpha tubulin (T5168, Sigma Aldrich, US) and anti-Ku80 (C48E7, Cell Signaling Technology, US) antibodies. All antibodies were diluted in 1% (w/v) non-fat milk powder in TBS–Tween. Proteins were visualized using enhanced chemiluminescence (ECL RevelBlot Plus or Intense, Ozyme, France) depending on the intensity of the signal. Protein lysate isolated from Saos-2 p53-null cell line engineered to over-express specific p53 isoforms cDNA were used as reference for p53 isoforms identification.

### Estimation of tetramer formation probability

As Delta40p53 and Delta133p53 isoforms are able to dimerize with TAp53, the probability of forming tetramers containing different configuration of DeltaNp53/TAp53 subunits were calculated using the formula developed by Chan et al. (2004) to estimate the composition of p53 oligomers [16]. Briefly, the ratio of p53 isoform transcript (Delta40p53, Delta133p53 and p53 beta) to full-length p53 protein, *r* was calculated in each sample using the formula (E_isoform_^Cq isoform^/E_TAp53_^CqTAp53^), in which E denotes the amplification efficiency of the transcript-specific primers. Separate pairwise estimates were calculated for TAp53-Delta40p53 and TAp53-Delta133p53. No attempt was made at estimating complexes including more than 2 isoforms.

### Statistical analysis

Statistical analysis and figures were generated with R studio version (1.1.463) and GraphPad Prism 7.00 software (GraphPad, San Diego, CA). One-way ANOVA followed by Benjamini-Hochberg multiple correction test was applied for comparisons across 2 or more groups. Data were tested at a statistical significance level of *p* < 0.05 and expressed as median ± interquartile range.

## Results

### Expression of p53 mRNA variants in primary and relapse BCP-ALL

**Fig. 1:**
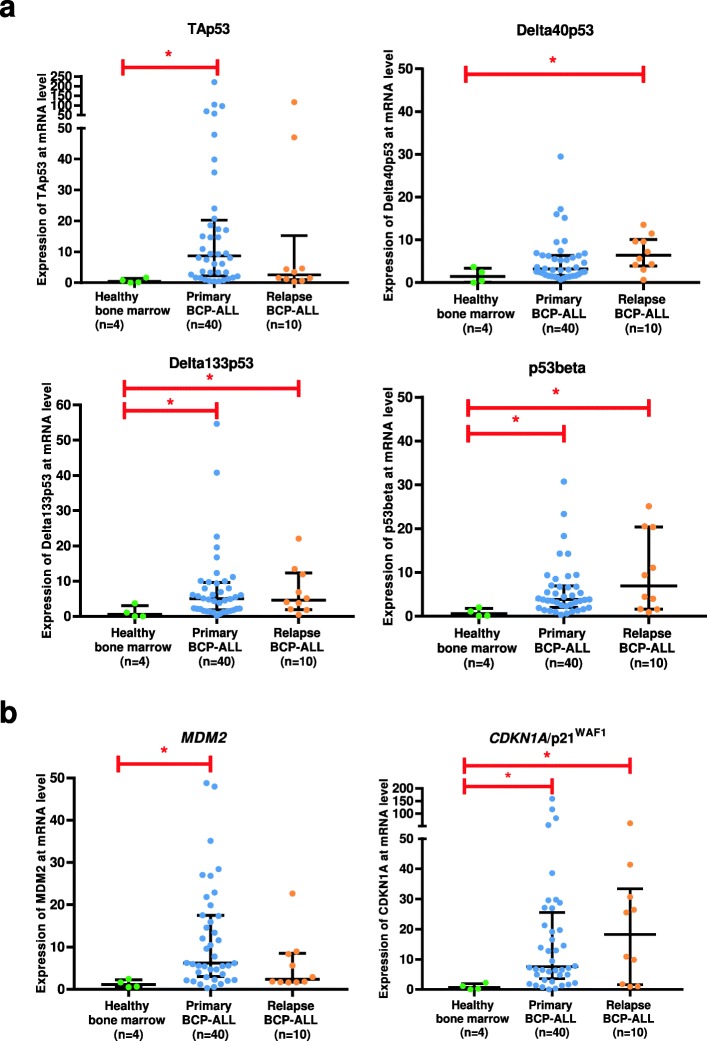
Semi-quantitative PCR analysis of p53 mRNA variants and p53-target genes in controls, primary and relapse BCP-ALL. **a** TAp53, Delta40p53, Delta133p53 and p53beta variants (**b**) *MDM2* and *CDKN1A* (p21^WAF1^). * denotes significant *p*-value (*p* < 0.05) calculated using One-way ANOVA with Benjamini-Hochberg correction. Bars represent median and interquartile range

The mRNA variants encoding p53 isoforms TAp53, Delta40p53, Delta133p53, and p53 beta were analysed in BMMC from 4 healthy donors, 40 patients with primary BCP-ALL and 10 patients with relapse BCP-ALL (demographic and cytogenetic profiles summarized in Table 1). We found different, contrasting trends in the expression levels of these transcripts in the three conditions (Fig. 1a). Irrespective of isoforms, levels of p53 mRNAs were low in BMMC from healthy donors. In primary BCP-ALL, TAp53 mRNA showed a median fold-induction of about 8.66, which was not seen in relapse BCP-ALL. In contrast, the median fold-induction level of Delta40p53 was increased in relapse BCP-ALL, while levels of Delta133p53 mRNA were significantly increased in both primary and relapse BCP-ALL. Likewise, p53beta mRNA showed a significant trend for increased expression in both primary and relapse BCP-ALL. Semi-quantitative evaluation of the expression of two bona fide p53 target genes, *MDM2* and *CDKN1A* revealed that both transcripts were markedly increased in primary BCP-ALL as compared to healthy donors, whereas only *CDKN1A* mRNA but not *MDM2* was increased in relapse BCP-ALL (Fig. 1b). Overall, these results suggest that changes in the expression of p53 isoforms occur in BMMC from primary and relapse BCP-ALL patients as compared to healthy donors. In primary BCP-ALL, patterns of p53 isoform mRNAs were dominated by increased levels of mRNA encoding TAp53, with concomitant detectable increase in both *CDKN1A* and *MDM2* mRNAs. In relapse BCP-ALL, predominantly expressed isoforms were Delta40p53, Delta133p53 and p53beta, and this was associated with increased levels of only *CDKN1A*, but not *MDM2* mRNA.

**Table 1:** Demographic and cytogenetic features of the study cohort

Variable	Group: No. (%) or Median [IQR] ^a^		
	Primary BCP-ALL	Relapse BCP-ALL^b^	Controls
Total no. in each group	40	10	4
Gender			
Male	24 (60)	5 (50)	2 (50)
Female	16 (40)	5 (50)	2 (50)
Age at recruitment, year	5.8 (3.1–8.2)	9.7 (5.2–10.9)	15.7 (12.3–19.6)
Cytogenetic features			NA
*BCR-ABL1*	10 (25)	1 (10)	
Hyperdiploid	10 (25)	0 (0)	
*ETV6-RUNX1*	1 (3)	0 (0)	
*MLL* rearrangement	0 (0)	1 (10)	
Others^§^	19 (48)	5 (50)	

Abbreviation: *IQR* interquartile range, *NA* not applicable

^a^ Categorical variables are reported as number with percentages, and continuous variables are reported as median with IQRs

^b^Cytogenetic information of 3 relapse cases are not available

Subtype “Others^§^” denotes those negative for tested molecular/cytogenetic features

### Predicted impact of variant p53 mRNA expression on the formation of p53 protein tetramers

N- truncated p53 isoforms have been shown to exert dominant-negative effects on p53 transcriptional activity by competitively associating with full-length p53 proteins in hetero-tetramers through their conserved C-terminal oligomerization domains [17]. With Delta40p53, hetero-tetramer formation generates protein complexes that retain DNA-binding but have impaired transcriptional activity due to lack of N-terminal sequences that bind co-activators of transcription. Delta133p53, in contrast, lacks part of the DNA binding domain and thus forms hetero-tetramers that are impaired in their capacity to bind to p53-regulatory DNA elements. In both cases, these isoforms exert a dominant negative effect on p53 activity when present in excess to TAp53.

**Fig. 2:**
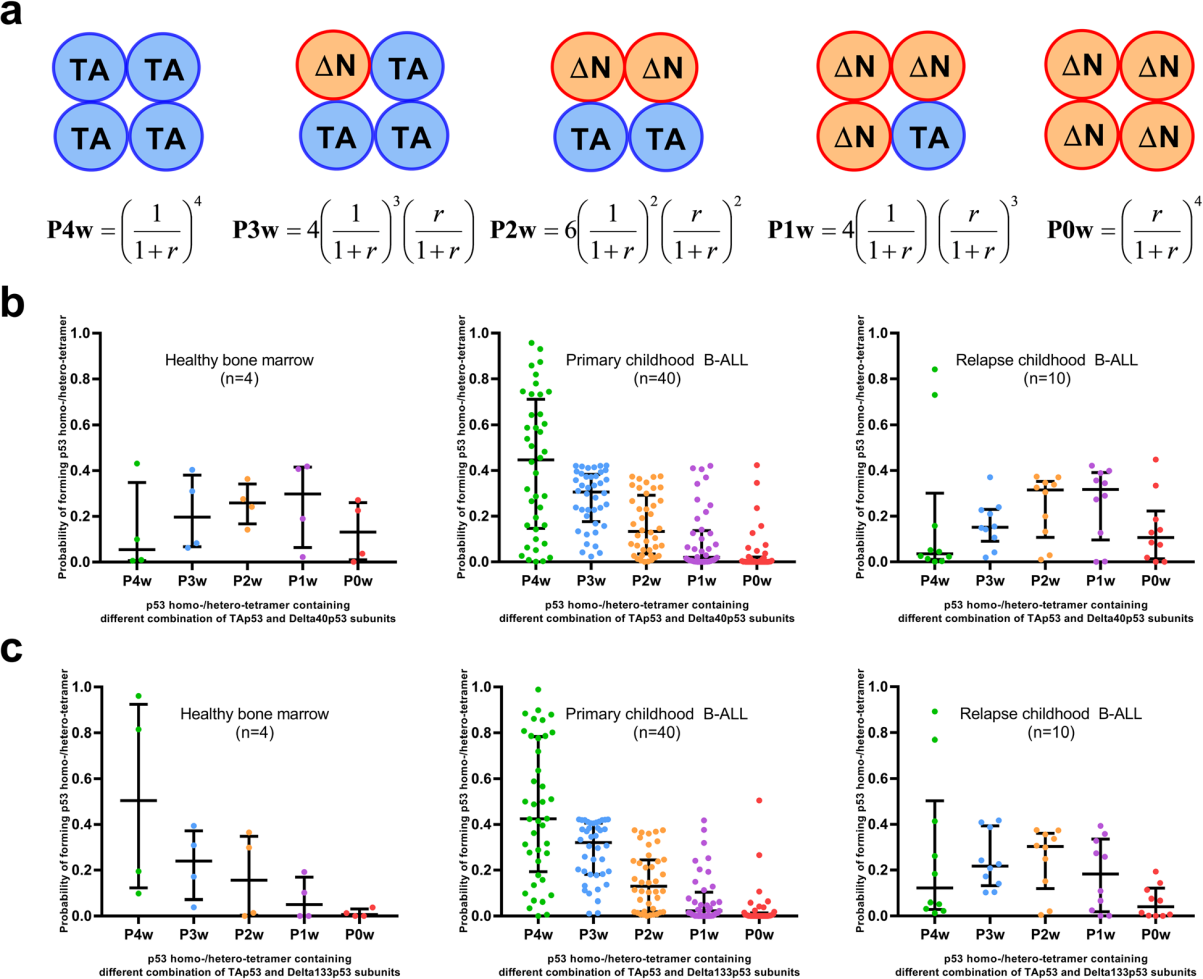
Probability of forming p53 homo- or hetero-tetramer in controls, primary and relapse BCP-ALL. **a** Mathematical calculation to predict probability of forming p53 tetramer containing different numbers of TAp53 and N-terminal p53 isoforms, using the formulae adopted from Chan et al. [16]. In relation with expression levels of N-terminal p53 variants, probability of forming (**b**) Delta40/TAp53 tetramer and (**c**) Delta133/TAp53 tetramer were determined in each subject. Bars represent median and interquartile range distribution

To evaluate whether the observed levels of p53 variant mRNAs had a dominant effect over TAp53, we estimated the probability of formation of different oligomeric configurations of p53, assuming equal translation of each mRNA variant, equal stability of each p53 isoform and equal incorporation rates into p53 tetramers (Fig. 2a). Our findings suggest that the rate of formation of different p53 tetramers may largely differ between BMMC from healthy donors, primary and relapse BCP-ALL patients (Fig. 2b and c). For TAp53-Delta40p53 complexes, the main predicted configurations were hetero-oligomers containing one to three TAp53 and Delta40 p53 in BMMC from healthy donors (Fig. 2b). In primary BCP-ALL, the main configuration was TAp53 homo-tetramers. In relapse BCP-ALL, however, the predicted configurations appeared similar to the one observed in BMMC from healthy donors. Results for TAp53-Delta133p53 complexes were essentially similar, the main difference being a higher predicted proportion of TAp53 homo-oligomers in BMMC from healthy donors (Fig. 2c).

Next, we assumed that p53 oligomers containing ≥3 TAp53 would be essentially transcriptionally active and those with ≥3 N-terminally truncated isoforms would be essentially inactive, whereas those with balanced levels of different isoforms may have an intermediate level of activity (Table 2). In the majority of primary BCP-ALL, the predicted configurations were active, whereas the majority of healthy donors and patients with relapse BCP-ALL had predicted inactive or intermediate configurations. Among different molecular and clinical subtypes of BCP-ALL, hyperdiploid BCP-ALL exclusively showed predicted active configurations, whereas 6/10 *BCR-ABL1* BCP-ALL had predicted intermediate or inactive configurations. These observations suggest that levels of competitively interacting p53 isoforms and, consequently, configurations of p53 tetramers, may significantly differ in relation to molecular and/or clinical characteristic (Table 2).

**Table 2:** Number of subjects with predicted transcriptionally active, intermediate or inactive p53 tetramer in each subgroup

	Predicted activity of p53 tetramer based on DeltaNp53:TAp53 expression ratio					
	Delta40p53:TAp53			Delta133p53:TAp53		
	ACT (P4w + P3w > 0.5)	INT	INACT (P1w + P0w > 0.5)	ACT (P4w + P3w > 0.5)	INT	INACT (P1w + P0w > 0.5)
Control (*n* = 4)	1	1	2	3	1	0
Primary BCP-ALL (*n* = 40)	30	6	4	32	6	2
hyperdiploid	10	0	0	10	0	0
*BCR-ABL1*	4	3	3	4	4	2
*ETV6-RUNX1*	1	0	0	1	0	0
Others^§^	15	3	1	17	2	0
Relapse BCP-ALL (*n* = 10)	3	4	3	5	3	2

Based on the calculated probability of forming different DeltaNp53:TAp53 oligomeric configurations (P0w-P4w), the transcriptional activity of p53 tetramer in each subject was predicted

*ACT* active, *INT* intermediate, *INACT* inactive

Subtype “Others^§^” denotes those negative for tested molecular/cytogenetic features

### Predicted ratio of p53 beta to N-terminal p53 isoforms

**Fig. 3:**
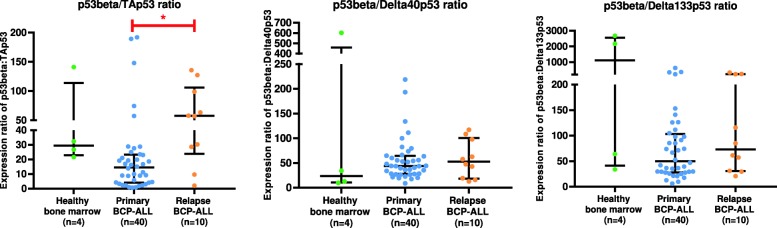
Expression ratio of p53beta in relative to N-terminal p53 variants in controls, primary and relapse BCP-ALL. * denotes significant p-value (*p* < 0.05) calculated using One-way ANOVA with Benjamini-Hochberg correction. Bars represent median and interquartile range

**Fig. 4:**
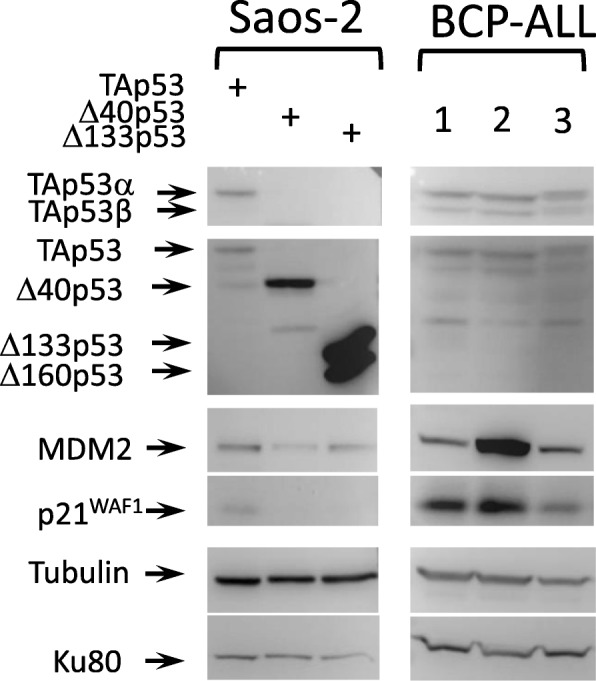
Expression patterns of p53 isoforms and p53 downstream effectors at protein levels in BCP-ALL. Representative western blot images showing expression of various p53 isoform proteins, MDM2 and p21^WIP^ in diagnostic bone marrow isolated from BCP-ALL patients (1: *BCR-ABL1*-positive primary BCP-ALL, 2: hyperdiploid primary BCP-ALL, 3: relapsed BCP-ALL). Control Saos-2 cell lines engineered to overexpress specific p53 isoforms were used as reference for p53 isoforms identification. Alpha-tubulin and Ku80 were used as loading controls. The images shown here are cropped and the full-length original blots are shown in Additional file 2

The p53beta isoform lacks the C-terminal oligomerization domain and is thus predicted to lack the specific, high-affinity DNA binding and transactivation activity that characterizes tetrameric TAp53alpha [17]. Assuming equal rates of expression and equal protein stabilities, the ratio of p53beta to all 3 N-terminal variants is strongly skewed towards p53beta in all three subgroups, with a significantly increased ratio of p53beta/TAp53 in relapse versus primary BCP-ALL patients (Fig. 3). Western blot analysis of bone marrow cells from patients confirmed that p53beta was one of the major p53 isoforms detected in BCP-ALL (Fig. 4), whereas the p53 protein was barely detectable in cells from healthy donors.

## Discussion

Mutations in *TP53* are infrequent in primary BCP-ALL. In a study of 240 BCP-ALL by Ding et al., *TP53* mutations were only observed in 5 cases [3]; and no genomic gain or loss of TP53 was detected in another independent cohort of 192 BCP-ALL [18]. This low *TP53* mutation frequency in BCP-ALL is not duplicated in any other hematopoietic malignancy or solid tumours [19]. Given the wide functions of p53 in tumour suppression, it is tempting to speculate that BCP-ALL cells may have developed strategies other than mutation to circumvent p53 functions. In this study, we analysed the patterns of expression of mRNA encoding dominant-negative isoforms of the p53 protein in BMMC from 40 primary, 10 relapse BCP-ALL and 4 healthy donors. We focused our analysis on mRNAs encoding either TAp53 (transactivation-competent) or isoforms that lack the N-terminal domain, Delta40p53 and Delta133p53, for which there is consistent in vitro evidence of dominant-negative effect on the transactivation of target genes by TAp53. We also investigated the expression of mRNA encoding p53beta, which has been shown to regulate senescence in peripheral T-lymphocytes [15]. Our findings demonstrate that, irrespective of the isoform, levels of p53 mRNA are low in bone marrow cells of healthy donors (controls) but are increased by 2 to 20-fold in primary or relapse BCP-ALL compared to controls. Specifically, TAp53 and Delta40p53 are increased in primary and relapse BCP-ALL, respectively, whereas Delta133p53 and p53beta are increased in both.

Using mRNA levels as a basis to infer the ratio between different protein levels in controls, primary or relapse BCP-ALL, our results show that, in primary BCP-ALL, p53 predominantly exists in oligomeric conformation dominated by TAp53. In contrast, in relapse BCP-ALL, p53 mostly exists in conformations that incorporate ≥2 Delta40 or Delta133p53 isoforms. We interpret these results as an indication that, in relapse BCP-ALL, p53 activity is functionally attenuated by competition with dominant-negative isoforms, whereas in primary BCP-ALL, p53 appears to be mostly made of TA homo-oligomers capable of transactivation. In controls, mRNA levels are extremely low and relative expression levels of isoforms appear to favour the formation of heterogeneous isoforms. These results are compatible with the hypothesis that deregulation of p53 isoform patterns may contribute to downregulate p53 functions, at least in relapse BCP-ALL. The p53beta isoform appeared to be overexpressed by about 6- fold in primary BCP-ALL and by about 11-fold in relapse BCP-ALL. When comparing the ratio of different p53 mRNAs, p53beta mRNA levels were significantly higher in relapse than in primary BCP-ALL. As a note of caution, it should be noted that these predictions rely on several hypotheses that need to be fully substantiated, namely, that different variant mRNAs are translated with the same efficiency and that the resulting p53 isoforms have similar cell distribution and stability. It is known that N-terminal isoforms particularly Delta40p53 could also be generated by internal initiation at AUG40 using TAp53 mRNA and has a higher stability than full-length p53. Thus, in relapse BCP-ALL, the effect of proposed p53 inactivity secondary to high level of Delta40p53 mRNA may be augmented by a combination of greater stability and increased production of Delta40p53 protein from alternative translation [20, 21]. The analysis of *CDKN1A* and *MDM2* mRNA levels are consistent with the hypothesis that p53 may be predominantly in an active form in primary BCP-ALL as levels of both mRNA are significantly increased over controls. In contrast, in relapse BCP-ALL, only levels of *CDKN1A* are increased as compared to controls. Whilst both genes can be upregulated through p53-independent pathways, their levels of expression may possibly reflect the state of p53 activity in these cells.

Taken together, these results suggest that expression of p53 isoforms is deregulated in BCP-ALL, with patterns of expression compatible with active TAp53 isoforms in primary BCP-ALL and with inactivation of p53 by alternative isoforms in relapse BCP-ALL. This is supported by Western blot analysis, which demonstrates that p53beta is a major protein isoform in BCP-ALL. Of note, in these patient-derived cells, patterns of N-terminally truncated p53 isoforms appear extremely complex and heterogeneous as compared with controlled cells engineered to overexpress specific p53 isoforms, making it difficult to unambiguously detect Delta40p53 and Delta133p53.

The precise molecular mechanisms by which isoform expression may subvert p53 functions are still a matter of conjecture. Delta133p53 and p53beta have been previously shown to play opposite roles in the regulation of cell senescence [22, 23]. Compared to proliferating fibroblasts, spontaneously senescent cells express increased levels of p53beta and decreased levels of Delta133p53. Conversely, expression of Delta133p53 is high in embryonic stem cells and induced pluripotent stem cells and appears to play a role in maintaining their high proliferative capacity [24]. In contrast with these results, relapse BCP-ALL show an ambiguous status, with increased levels of both Delta133p53 and p53beta mRNA. Sample-by-sample comparison shows that the levels of the two forms are actually not correlated with each other, suggesting that they are regulated independently. An interesting hypothesis is that relapse BCP-ALL could display states of chemotherapy-induced senescence that may actually enhance stem-cell related properties. Indeed, in a recent study by Milanovic et al. [25] using genetically switchable models of senescence targeting p53 and H3K9me, mouse B-lymphoma cells released from senescence were shown to re-enter the cell-cycle with strongly enhanced clonogenic growth potential, compared to cell populations which did not undergo senescence after chemotherapy. These results support the possible role of p53 in the control of stemness-associated senescence, a cancer cell phenotype that has major implications for understanding the mechanisms of relapse and resistance to therapy. The presence p53 isoform signatures of both stemness and senescence in relapse BCP-ALL may be an indicator of this specific phenotype.

In our study, semi-quantitative RT-PCR using primers specific to either the N- or C-terminal variations was used to detect relative differences in the mRNA expression of the N- and C-terminal variants Using this approach, we were unable to differentiate between the possible variants which result from unique combinations of N- and C-terminal variations (ie: TAp53alpha, TAp53beta, Delta40p53alpha, Delta40p53beta, Delta133p53alpha and Delta133p53beta) due to restrictions in amplicon size of 100-150 bp in semi-quantitative RT-PCR. In this study, the state of p53 activity in BCP-ALL was predicted based on the concept of p53 isoforms’ dominant negative effect over full-length p53 in this study. These results must be interpreted with caution as DeltaNp53 may also modulate p53 responses in a p53-independent manner [12]. Another limitation of our study is the small number of healthy controls and lack of matched samples collected at diagnosis and relapse, which would allow us to monitor if changes observed in relapses are de novo or following their primary cancers. In addition, this single-centre study only included selected representative subtypes of BCP-ALL namely that of good prognosis (hyperdiploidy), poor prognosis (*BCR-ABL1*) and others without a known cytogenetic feature, hence potentially limiting the generalisability to other BCP-ALL patients. The latter group could be extremely heterogeneous and include various prognostic subtypes. Further prospective studies using samples with complete genomic profile along with investigation of a larger set of high-confidence p53 target genes would allow us to reach more definite conclusions. Nevertheless, our findings provide some critical insights on the relative expression of different p53 isoforms in normal bone marrow, primary and relapse BCP-ALL.

## Conclusion

This study demonstrated that expression of p53 isoforms is deregulated in BCP-ALL, with expression patterns leaning towards alternative forms of p53 in relapse BCP-ALL. Variations in p53 isoform expression may contribute to a mechanism of p53 protein inactivation in BCP-ALL, specifically contributing to the poor prognosis in relapse forms.

## Supplementary information

**Additional file 1.** Primer sequences for real time RT-PCR and schematic diagram of human p53 isoforms.

**Additional file 2.** Full-length original blots of Fig. 4

## Data Availability

The data that support the findings of this study are available from the corresponding author on reasonable request.

## Abbreviations

BCP-ALL: B-cell precursor acute lymphoblastic leukemia
BMMC: Bone marrow mononuclear cells
RT-PCR: Reverse-transcriptase PCR
