# Supplementary material for: Expression of p53 N-terminal isoforms in B-cell precursor acute lymphoblastic leukemia and its correlation with clinicopathological profiles
Source: BMC Cancer. 2020 Feb 10;20:110. doi: 10.1186/s12885-020-6599-8 (PMC7011217; doi:10.1186/s12885-020-6599-8)

**Additional File 1: Primer sequences for real time RT-PCR and schematic diagram of human p53 isoforms.**


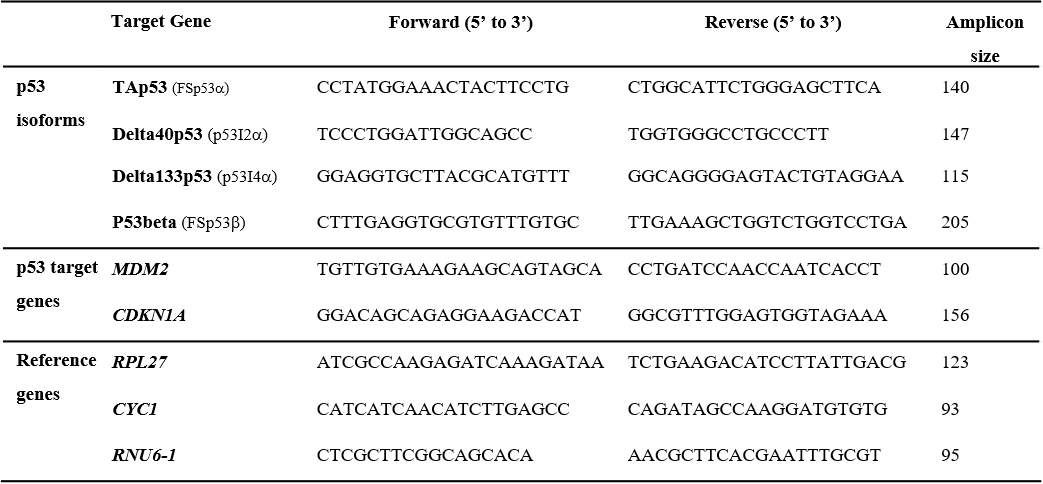
Table showing primer sequences used in real-time RT-PCR and schematic diagram showing genetic and structural organization of human p53 isoforms along with location of primers specific for each *TP53* mRNA variants.


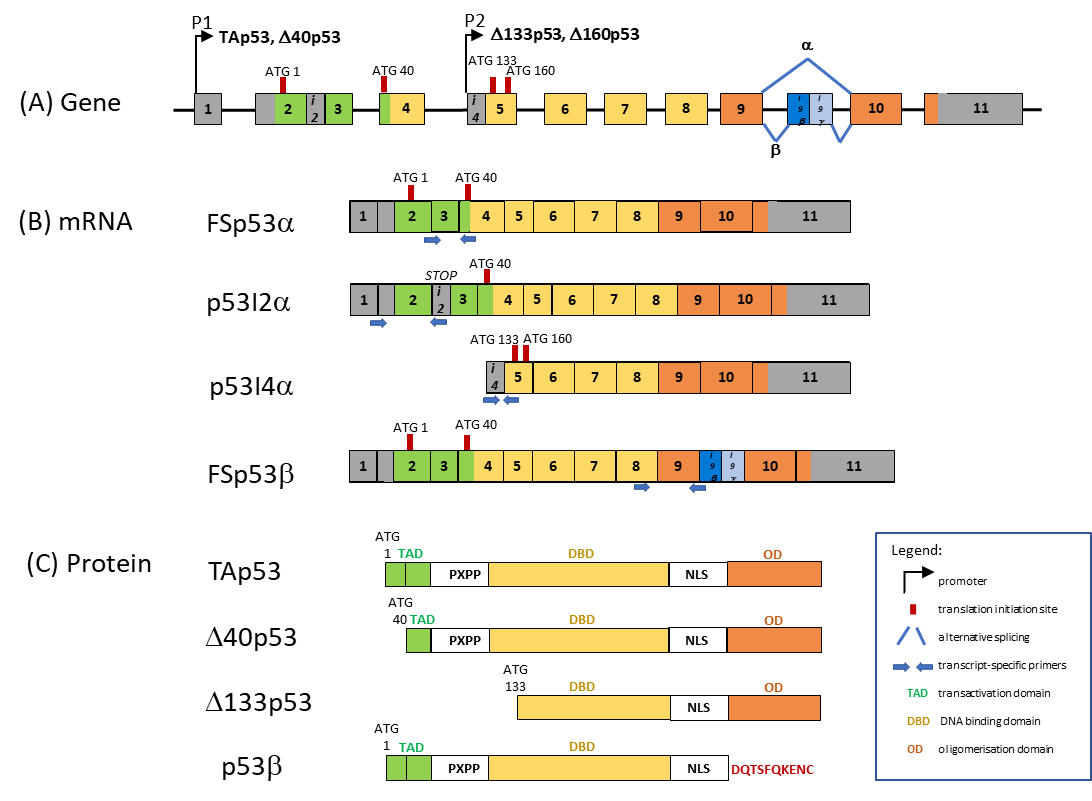

Supplement: Supplementary file 1 — Additional file 1. Primer sequences for real time RT-PCR and schematic diagram of human p53 isoforms. [file 12885_2020_6599_MOESM1_ESM.docx]
