# Supplementary material for: Expression of p53 N-terminal isoforms in B-cell precursor acute lymphoblastic leukemia and its correlation with clinicopathological profiles
Source: BMC Cancer. 2020 Feb 10;20:110. doi: 10.1186/s12885-020-6599-8 (PMC7011217; doi:10.1186/s12885-020-6599-8)

**Additional File 2: Full-length original blots of Figure 4**

Protein extracted from diagnostic bone marrow of BCP-ALL patients (Lane 2: *BCR-ABL1*-positive primary BCP-ALL, Lane 3: hyperdiploid primary BCP-ALL, Lane 4: relapsed BCP-ALL) and control Saos-2 cell lines overexpressing specific p53 isoforms (Lane 13: TAp53alpha, Lane 14: Delta40p53alpha, Lane 15: Delta133p53alpha) were ran in two identical sets and transferred to PVDF membranes.

Membrane 1 and membrane 2 were cut into two parts. Upper part of membrane 1 was blotted with (a) anti-Ku80 antibodies, while bottom part of the membrane was blotted sequentially with (b) 9282 antibodies followed by (c) DO-7 antibodies. Similarly, upper part of membrane 2 was blotted sequentially with (d) anti-MDM2 antibodies followed by (e) anti-alpha tubulin antibodies while bottom part of membrane 2 was blotted with anti- p21^WAF-1^ antibodies.

(a)


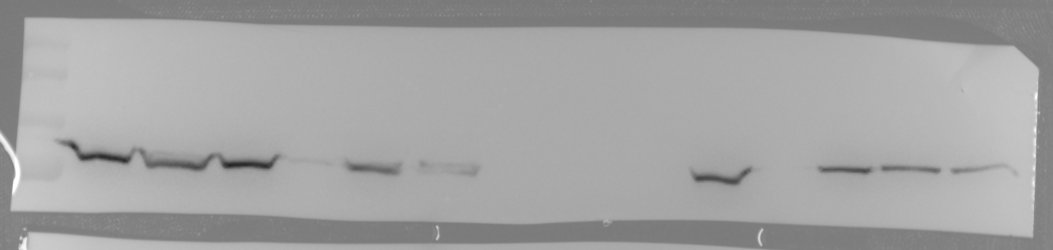


(b)


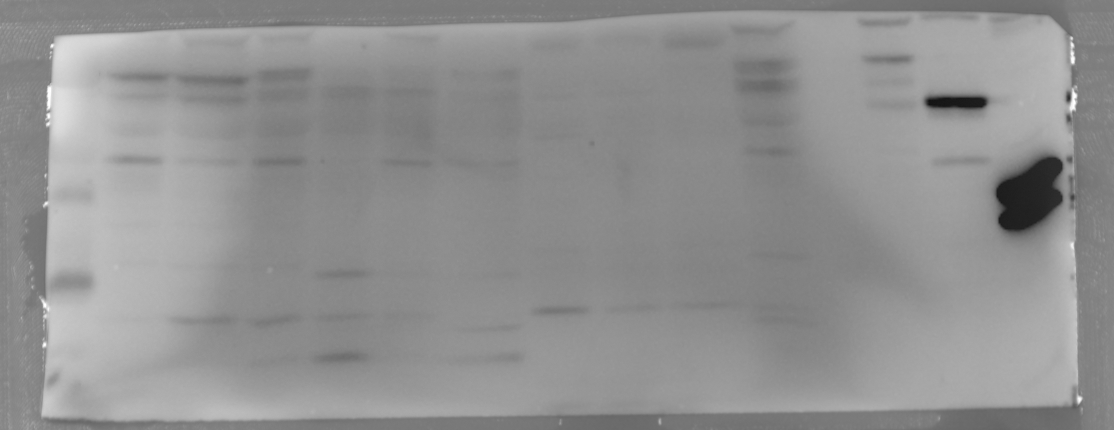


(c)


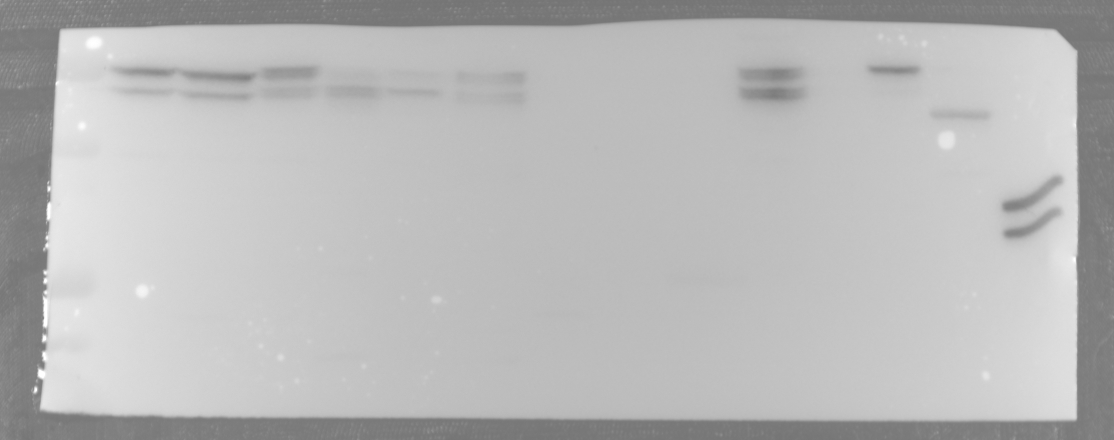


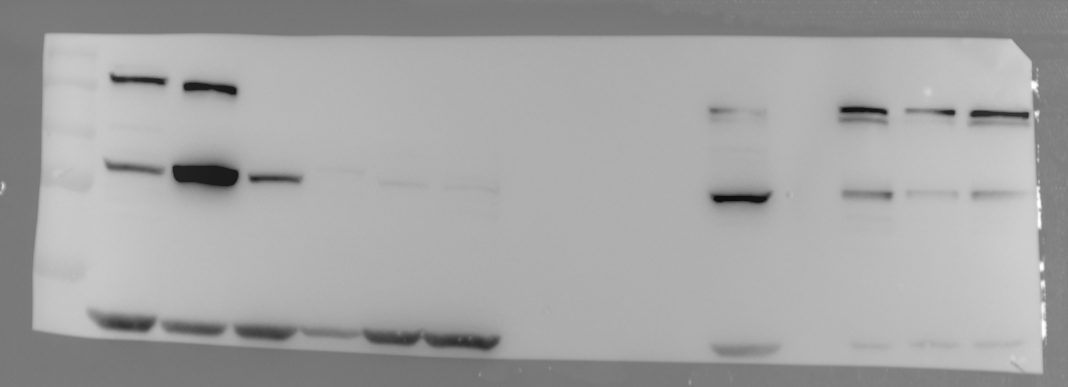


(e)


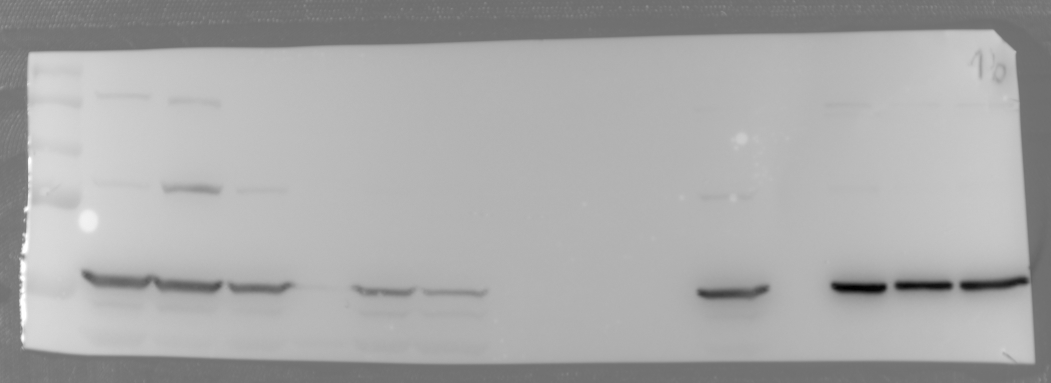


(f)


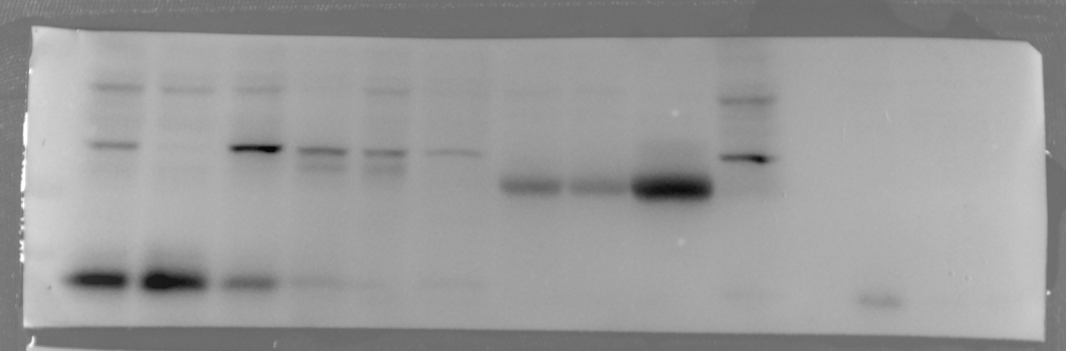

Supplement: Supplementary file 2 — Additional file 2. Full-length original blots of Fig. 4 [file 12885_2020_6599_MOESM2_ESM.docx]
